# A Versatile Approach for Adaptive Grid Mapping and Grid Flex-Graph Exploration with a Field-Programmable Gate Array-Based Robot Using Hardware Schemes

**DOI:** 10.3390/s24092775

**Published:** 2024-04-26

**Authors:** Mudasar Basha, Munuswamy Siva Kumar, Mangali Chinna Chinnaiah, Siew-Kei Lam, Thambipillai Srikanthan, Gaddam Divya Vani, Narambhatla Janardhan, Dodde Hari Krishna, Sanjay Dubey

**Affiliations:** 1 Department of Electronics and Communication Engineering, Koneru Lakshmaiah Education Foundation, Green Fields, Guntur 522502, Andhra Pradesh, India; mudasar.basha@bvrit.ac.in (M.B.); msivakumar@kluniversity.in (M.S.K.); 2 Department of Electronics and Communications Engineering, B. V. Raju Institute of Technology, Medak (Dist), Narsapur 502313, Telangana, India; divyavani.g@bvrit.ac.in (G.D.V.); harikrishna.dodde@bvrit.ac.in (D.H.K.); sanjay.dubey@bvrit.ac.in (S.D.); 3 School of Computer Science and Engineering, Nanyang Technological University, Singapore 639798, Singapore; ASSKLam@ntu.edu.sg (S.-K.L.); astsrikan@ntu.edu.sg (T.S.); 4 Department of Mechanical Engineering, Chaitanya Bharati Institute of Technology, Gandipet, Hyderabad 500075, Telangana, India; njanardhan_mech@cbit.ac.in

**Keywords:** single robot, robotic exploration, tree and graph structure, hierarchical mapping, FPGA, grid flex-graph exploration

## Abstract

Robotic exploration in dynamic and complex environments requires advanced adaptive mapping strategies to ensure accurate representation of the environments. This paper introduces an innovative grid flex-graph exploration (GFGE) algorithm designed for single-robot mapping. This hardware-scheme-based algorithm leverages a combination of quad-grid and graph structures to enhance the efficiency of both local and global mapping implemented on a field-programmable gate array (FPGA). This novel research work involved using sensor fusion to analyze a robot’s behavior and flexibility in the presence of static and dynamic objects. A behavior-based grid construction algorithm was proposed for the construction of a quad-grid that represents the occupancy of frontier cells. The selection of the next exploration target in a graph-like structure was proposed using partial reconfiguration-based frontier-graph exploration approaches. The complete exploration method handles the data when updating the local map to optimize the redundant exploration of previously explored nodes. Together, the exploration handles the quadtree-like structure efficiently under dynamic and uncertain conditions with a parallel processing architecture. Integrating several algorithms into indoor robotics was a complex process, and a Xilinx-based partial reconfiguration approach was used to prevent computing difficulties when running many algorithms simultaneously. These algorithms were developed, simulated, and synthesized using the Verilog hardware description language on Zynq SoC. Experiments were carried out utilizing a robot based on a field-programmable gate array (FPGA), and the resource utilization and power consumption of the device were analyzed.

## 1. Introduction

The fundamental challenge of accurately mapping and navigating complicated surroundings using a single robot requires innovative approaches. The global robotics market is expected to experience significant revenue growth, with service robotics having the highest share. This industry is projected to reach a volume of USD 65.59 billion by 2028 [1]. Robotic research focuses on the ability of mobile autonomous robots to map, explore, and locate themselves on an environmental map. Technologies such as Wi-Fi, Bluetooth, LiDAR, and computer vision are used to validate this exploration [2].

Over the past three decades, researchers have significantly advanced robotics, honing adaptation, visualization, navigation, sensing, communication, collision avoidance, and behavioral control algorithms on computational platforms to tackle exploration tasks with efficiency [3]. Exploration techniques begin by leveraging real-time range sensor data to ensure comprehensive coverage. Various methods like occupancy grid maps, feature map types, and topological feature map approaches drive autonomous exploration [4,5]. For instance, Wattanavekin et al. devised an indoor mapping system, integrating environmental boundary information, Reactive Diffusion Equation on Graph (RDEG) integration, labeling, and a re-planning framework using infrared sensors and ultrasonic beacons for grid-based representations [6,7]. Meanwhile, Gao et al. pioneered a 2D laser-based autonomous exploration method, although it faced challenges in adapting to dynamic environments [8]. Wang et al. optimized an algorithm addressing the loop-closing problem in embedded topological worlds, highlighting tradeoffs between computational and robot traversal costs [9]. Their integrated approach identifies grid cells to simulate state occupancy and employs frontier-based graph exploration akin to breadth-first search (BFS) to determine the robot traversal direction thanks to grid mapping, which divides the environment into identical dimensions for easy access to cells and data.

Frontier-based graph exploration has emerged as a strategic technique for understanding the environment, starting from a designated point and expanding outward. Researchers have proposed diverse strategies for autonomous robot exploration in unknown territories. For example, Wirth et al. introduced a breadth-first search (BFS) approach, prioritizing exploration and search tasks [10]. Yu et al. suggested a dual RGB-D sensor system for mapping unknown indoor spaces, tackling computational hurdles [11]. Argenziano et al. put forth a graph-based robot representation integrating sensor readings with semantic understanding [12]. Dudek et al. honed robotic exploration in graph-like worlds using markers and heuristic methods [13]. Perea et al. devised a method leveraging a map database and predicting environment expansion [14]. Niroui et al. introduced a Partially Observable Markov Decision Process for exploration, focusing on mapping and object avoidance [15,16,17]. Rojas et al. developed mapping and navigation algorithms employing Fast SLAM, Vector Field Histogram, and spiral way trajectory methods [18]. Mikko Lauri introduced a sample-based approximation for mutual information in mobile robotics, which was integrated with forward simulation planning algorithms [19]. Meanwhile, Basilico et al. suggested a multicriteria decision-making approach [20]. Gomez et al. introduced a frontier using a behavior-based concept [21]. Yamauchi et al. pioneered Autonomous Robot for Integrated Exploration and Localization (ARIEL) system mapping [22]. The author presents an evolvable hardware-based autonomous robot navigation system, utilizing intrinsic evolution and evolutionary computing for hardware learning to enhance grid-based representation effectiveness in real-time scenarios [23]. Mingas et al. proposed the SMG-SLAM algorithm for resource-efficient mapping in unfamiliar environments [24]. Al-Ansarry et al. proposed path planning method which merges dijkstra algorithm with potential field collision avoidance for safe path planning in real time dynamic environments [25]. Song et al. devised a structure-based spatial exploration system employing machine learning and artificial intelligence [26]. Episodic memory-based topological mapping (e-TM) offers a neural network framework using sparser topological graphs to model exploration trajectories as episodic memory for mobile robot navigation [27]. The authors propose a new path-planning algorithm based on the chaotic behavior of the Courbage–Nekorkin neuron model, utilizing a pseudorandom bit generator (PRBG) to create a motion trajectory in four or eight directions and minimizing repetitions [28,29].

However, the challenge of effective exploration, path planning, and mapping, coupled with high computational costs, persists in indoor environments during exploration tasks. Our proposed approach, the grid flex-graph exploration technique, introduces grid and graph flexibility structures, offering the optimal solution for the dual-level depiction of the environment by leveraging an FPGA hardware accelerator for real-time applications.

The following are the novelties of the grid flex-graph exploration approach:

1. Hardware-based behavior using quad-grid construction was developed for the exploration algorithm.
2. Distance-based frontier detection and local-occupancy-based frontier detection graph algorithms with probabilistic mapping were developed with PR flow and hardware schemes.
3. Redundancy checks were developed for the complete exploration module of the environment to optimize local map data and dynamic graphs using hardware schemes.
4. A hardware-driven partial reconfiguration process was incorporated to execute multiple algorithms based on event-driven conditions.

This section provides a concise overview of robotic systems and the existing research on robot exploration. Our proposed methodologies using VLSI hardware schemes are presented in Section 2. A demonstration of the feasibility of the proposed method, supported by evidence such as device utilization, power consumption, empirical validation, and both qualitative and quantitative comparisons with alternative techniques, is presented in Section 3. Section 4 presents the strengths and potential of the proposed method.

## 2. Hardware-Based Algorithms

This section presents the proposed hardware-based techniques for grid flex-graph exploration in dynamic environments. The proposed abbreviations are shown in Table 1.

### 2.1. Algorithm for Exploring Grid Flex Graphs Using Hardware-Based Methods

**Figure 1:**
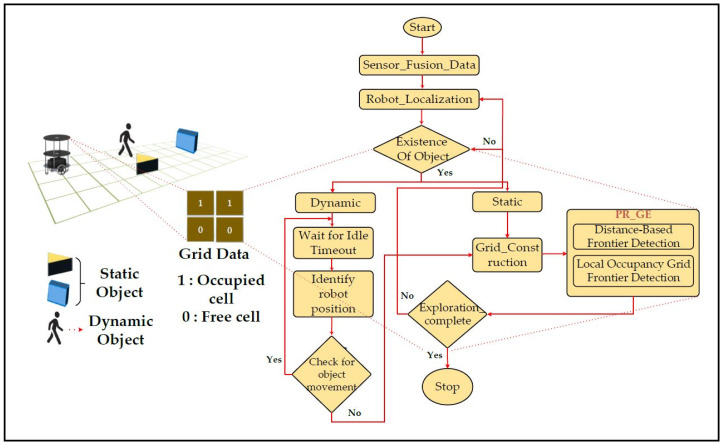
Flowchart of robot exploration in an indoor environment.

An overview of grid flex-graph exploration in an indoor environment that consists of static and dynamic objects is shown in Figure 1. Exploration involves integrating fused sensor data for the robot to localize in indoor environments. The robot begins to explore the environment in the presence of static or dynamic objects. The proposed system evaluates static objects that do not vary with time and dynamic objects that vary with time. In the presence of static objects, the robot begins to construct a grid with occupancy information and traversability characteristics. The quadtree divides the grid into quadrants (NW, NE, SW, and SE), allowing for a hierarchical structure in which cells at different levels represent varying levels of information. Concurrently, distance-based frontier detection and a local occupancy grid frontier detection graph approach are utilized to enhance spatial granularity. The robot navigates the environment, prioritizes frontiers based on their directions, and refines the spatial information provided by the quadtree structure. In the presence of dynamic objects, the robot utilizes the timing concept to confine the objects and the position of the robot. Once the robot has confined the dynamic objects, it utilizes the exploration process of continuously updating the quadtree-enhanced grid, capturing detailed information, and adapting its strategy based on a refined representation. The cyclical process of fused sensor data integration, quadtree grid construction, and partial reconfiguration-based frontier-graph exploration contributes to an adaptive and efficient robotic exploration approach is shown with red color dotted lines. Constraints must be considered when implementing the proposed strategy.

Robot Position Constraints: These constraints ensure that the coordinates (x, y, θ) of the robot remain within the grid boundaries (1 ≤ x*_i_* ≤ *M*, 1 ≤ y*_i_* ≤ *N*, *1* ≤ θ*_i_* ≤ *2π*).

Ultrasonic Sensor Reading Constraints: These constraints ensure that the ultrasonic sensor readings fall within the allowable range (*D*_min_ < *D_i_* < *D*_max_).

Grid Resolution Hypothesis: The grid resolution is sufficiently fine to capture relevant details in the environment.

Graph Connectivity Constraints: The graph representation must be binary to indicate whether the nodes are connected:

*G_ij_* ∈ {0, 1}. (1)

Objective Function: The objective is to maximize the sum of the occupancy values in the grid multiplied by the corresponding graph connectivity. The objective function is expressed as follows:

(2) $$Maximize\sum_{i=1}^{N}\sum_{j=1}^{M}M_{ij}\cdot G_{ij}.$$

This objective encourages the exploration and mapping of areas with objects and optimizes the graph for efficient navigation.

Graph Update: The graph is dynamically updated based on the closure detection between neighboring nodes in the graph.

$$G_{ij}=\left\{\begin{array}{l} 1\;\;\;\;\;\;\;\;\;\;\;\;\;\;\mathrm{If}\;\text{closure}\;\text{is}\;\text{detected}\;\text{between}\;\text{nodes}\left(i,j\right)\;\text{based}\;\text{on}\;\text{sensor}\;\text{readings}\\ 0\;\;\;\;\;\;\;\;\;\;\;\;\;\mathrm{Otherwise} \end{array}\right.$$

Exploration of Complete Detection: Complete detection checks whether the difference in the ultrasonic distances between two neighboring nodes is below a specified threshold and is given as

*D_i_*_1*j*1_ − *D_i_*_2*j*2_ < *C*_threshold_, (3)

where (*i*_1_, *j*_1_) and (*i*_2_, *j*_2_) are neighboring nodes in a graph.

#### 2.1.1. Exploring Grid-Based Graph Using a Hardware-Based Algorithm

This section explores a grid-based graph in an environment with both static and dynamic objects. Algorithm 1 represents the pseudocode of grid construction and graph exploration.

**Algorithm 1.** Pseudocode for grid construction and graph exploration.
1. Initialize R_S_ = {S_F_, S_L_, S_R_, S_B_}, S_D_ = {S_FR_, S_FL_, S_LB_, S_RB_}, R_P_ = {x_i_, y_i_, θ_i_}, E = {M × N}, R_D_, G_D_
2. Initialize F = {7{R × C}}, digital compass direction
3. Case_1 (Grid_Construction)
4. State1_1: if ((R_S_ == d_max_) && (S_D_ == dd_max_))?((R_S_ == d_min_) && (S_D_ == dd_min_))?
    State3_1@Update_map: State2_1@Graph_Exploration: State1_2;
5. State1_2: if ({S_F_, S_R_} > d_max_)? State_forward: State1_3;
6. State_forward: if (G_D_, R_D_ == d_max_)? (R_D_ == 90°): R@Fwd: R@Left: R@Right;
7. State1_3: State3_4@Update_map, repeat State_1
8. end case
9. Case_2 (Graph_Exploration)
10. State2_1: if ((node_queue () == 0)? ((R_S_ == D_max_) && (S_D_ == DD_max_))?   State1_1@Grid_Construction: State4_1@complete_exploration: State2_2;
11. State2_2: for (int i = 0; I ≤ n − 1; i++)      {       If (R_S_, S_D_ [i] ≤ D_max_) C_child_nodes[i] = C[i];      }
12. State2_3: if (((C1 == C2) ≤ D_max_) && (C4 == D_max_))       Robot Follow around the direction @turn_right, Odometer ++      else if (((C1 == C4) ≤ D_max_) && (C1 == D_max_))       Robot Follow along the direction
13. State2_4: if ((C1 == C2) ≥ D_max_) && (C4≤ Dd_max_)?(Time == 10)?1′b1:    State2_4@Graph_Exploration: State2_5;
14. State2_5: State3_2@Update_map, Repeat State2_1
15. end case


The pseudo code of Algorithm 1 represents a robotic exploration algorithm that employs a state-machine approach in a grid-based environment. Starting with lines 1 and 2, the initialization phase sets up essential parameters, such as sensor configurations, robot positioning with the help of a digital compass, environment size, and the initial map.

Subsequently, the pseudocode enters the quad-grid construction phase (Case_1) in line 3, where the robot checks the sensor readings against the maximum and minimum distances in the grid’s vicinity (d_max_ and d_min_) in line 4. The robot’s position in the environment determines whether to start with graph exploration or update the map in line 5. The movement of the robot is determined by sensor readings (R_S_ and S_D_) in different directions, and the algorithm repeats this process until the robot is positioned at the center node of the grid, as shown in lines 6–8.

**Figure 2:**
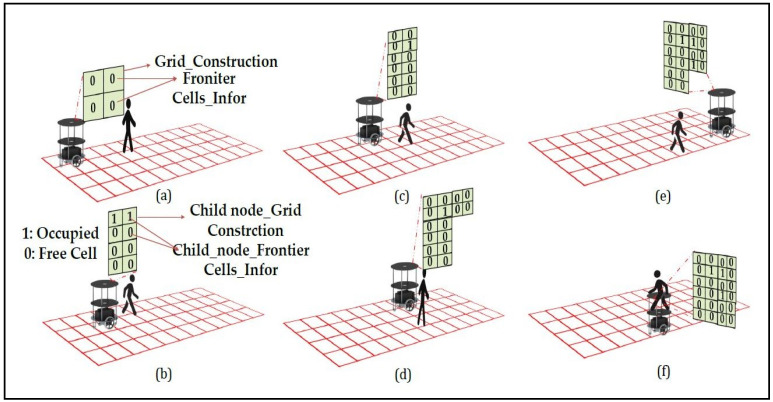
(**a**) Robot at initial grid. (**b**) Graph exploration by the robot. (**c**) Evaluation of dynamic object & traversing to next node. (**d**) Traversing based on cell information. (**e**) Confirming the dynamic object. (**f**) Avoiding dynamic object and completes the exploration by updating the map.

Figure 2a–f illustrates the robotic exploration of an indoor environment as shown in red color check boxes with dynamic objects. Figure 2a shows the robot is localized at the center position of the grid, it searches for neighboring nodes to start the graph exploration phase (Case_2) in line 9 to begin systematic exploration. The robot checks the node queue (line 10) and switches to the complete exploration in Algorithm 2 if the queue is empty, indicating that no neighboring child nodes are available for exploration. It then checks if the sensor readings are at their maximum distances (D_max_ and DD_max_) and decides whether to switch back to construct a new quad-grid or continue exploration based on the frontier direction. The algorithm uses specific sensor data to estimate a state that includes the posture of the robot and a representation of the surroundings. Figure 2b presents the exploration algorithm identifies frontier zones and selects the next destination based on the calculated information, as depicted in line 11. An important part of exploration involves choosing the next target efficiently based on priorities. Figure 2c,d presents the exploration priorities can be determined by examining the unexplored state and the adjacency of the nodes in the frontier-graph structure defined in line 12.

**Algorithm 2.** Pseudocode for updating map and complete exploration.
Initialize {FDmax, FD, FG, FV, FO, FMH}, Initialize {US, D, G, V, DO, PMH}Initialize {node, adjacency_list}Case_3 (Update_map)State3_1: if ((R_S_ == d_min_) && (S_D_ == dd_min_))? State4_1@complete_exploration:State3_2;State3_2: Update_map = {Store_Data in US_FIFO, Store_Data in Dir_FIFO, Store_Data in Grid_FIFO, Store_Data in Vertex_FIFO, Store_Data in DO_FIFO}: State3_3;State3_3: Map_merge = if (Previous_map_history == FMH)? Store_Data in History_FIFO: State3_4;State3_4: Global_map_FIFO = if ({FDmax == FD == FG == FV == FO == FMH == 1′b1}? 1′b1: Repeat State3_1;end caseCase_4 (complete_exploration)State4_1: if (a == adjacency_list(node(x))) && ((b == adjacency_list(node(y)))? State4_2: State4_3;State4_2: if ((node_position.x − child_position.x) == 0) && (node_position. y − child_position.y) == 0)? State3_2@Update_map: State4_3;State4_3: if ((node_position.x − sibling_position.x) > 1) && (node_position. y − sibiling_position.y) > 1)? State4_4: State3_2@Update_map;State4_4: if ((neighbor == queue.empty())? State3_1@Update_map: Repeat State4_1;end case.


The exploration algorithm chooses the closest unexplored child node following the exploration of the parent node or chooses the closest unexplored sibling node. The orientation and distance traveled of the robot are crucial for determining the subsequent target node. If the robot’s front and left halves have objects to identify, the robot moves in that direction by making 90° right turns to visit the sibling node; otherwise, the robot will move in the direction of the robot to visit the child node. An internal register is used to maintain the odometer count. Figure 2e shows that, the robot has to check for an idle timeout of 10 s to confine the dynamic objects, as shown in line 13. Figure 2f shows when the robot reaches an undiscovered node and identifies safe quad cells, we incorporate the newly discovered quad nodes to be child nodes in the existing frontier-graph structure. We update the node and local map information in line 14 and repeat the graph exploration process. The grid construction and graph exploration processes occur concurrently with the help of the proposed VLSI hardware’s internal architectures.

Exploration management is detailed in the complete exploration state (State4_1) in line 10 of Algorithm 2. Additionally, the update map state (State3_1, State3_2, and State3_4) is invoked in lines 4, 14, and 7 and switches to Algorithm 2 to update the corresponding quad-grid data, neighboring child and sibling nodes, and other information at time intervals based on sensor feedback. The robot’s movement is represented in Figure 2a–f. This update ensures that the map represents the environment accurately. This pseudocode encapsulates a comprehensive exploration strategy, transitioning among grid construction, graph exploration, complete exploration, and map updates based on real-time sensor data and environmental feedback.

**Figure 3:**
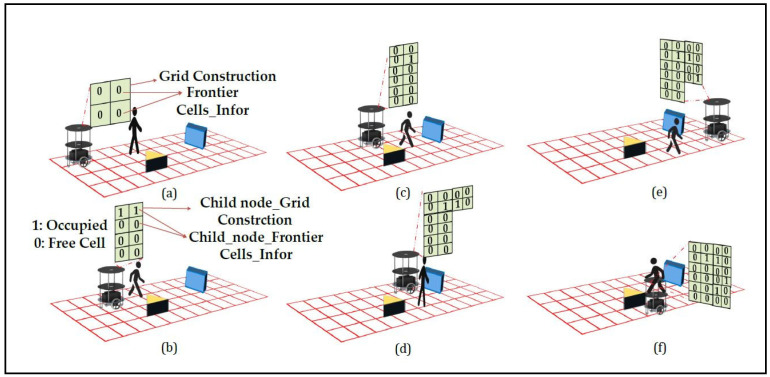
(**a**) Robot at initial grid. (**b**) Graph exploration by the robot. (**c**) Evaluation of static and dynamic object & traversing to next node. (**d**) Traversing based on cell information by confirming static and dynamic object. (**e**) Confirming the static and dynamic object. (**f**) Avoiding dynamic and static object based on cell information and completes the exploration by updating the map.

The pseudocode of Algorithm 2 presents a brief update to the map during robotic exploration, with a particular emphasis on complete exploration detection. Figure 3a–f illustrates the robotic exploration of an environment with static as blue color square box and yellow color triangle box and black color human as dynamic objects respectively. The initialization phase (lines 1–2) sets up arrays for the storage of sensor readings, distances, grid information, vertex information, detected object types, and map history. These data structures are crucial for decision making during the continuous exploration process, as illustrated in Figure 2a–f and Figure 3a–f. Figure 3a shows the algorithm leverages a case-based structure, where Case_3 (lines 3–8) focuses on updating the map based on the analyzed real-time data, indicating that the robot is at the center node of the grid. Figure 3b shows that the frontier cells are obtained from the existing local grid map. Figure 3c,d presents the exploration priorities of the unexplored state and the adjacency of the nodes. If the initial grid is the final grid, we conclude that the environment is closed, and there are no child or sibling vertices available for the robot to traverse in line 4. Hence, while storing data, it is feasible to recognize previously visited edges from the regional map that have been assessed and are recognizable areas in the local maps of adjacent nodes. We minimize the storage related to revisiting surrounding nodes in the graph by merging the local maps of the surrounding nodes to distinguish between the original frontier cells. Figure 3e shows that robot confines static and dynamic object with timeout enable feature and select cells that serve as frontiers on both the current local map and the merged map. Map merging can be achieved by utilizing parallel hardware architectures, which is a key novelty of the proposed approach, as stated in lines 5–7. The algorithm switches to Case_4 (lines 9–14), which signifies the complete exploration detection phase and is critical for maintaining map consistency. The algorithm checks whether two nodes (x_i_, y_i_) in the adjacency list correspond to the current robot position and evaluates their relative positions by comparing the reference data stored in internal registers a and b. If a potential completion is detected at line 10, it switches to updating the grid information and the neighboring vertex information. Otherwise, the algorithm performs further checks by moving to the next transition to determine whether a complete exploration should be confirmed or whether it should revert to the map update process. Figure 3f shows this mechanism, situated within the broader map-update algorithm, addresses the locations revisited during exploration, thereby enhancing the robustness and adaptability of the algorithm.

The complete exploration conditions (State4_2 and State4_3) in lines 11–12 consider the relative positions of the nodes in the adjacency list. The algorithm also checks for additional conditions (State4_4 and State4_5) to confirm complete exploration and perform subsequent actions. If a neighboring node is present in the queue, the algorithm repeats this process; otherwise, it transitions back to the map-update process in line 13. Updating the map with the help of grid information and traversing to the neighboring node are executed concurrently by switching from Algorithm 1 to Algorithm 2. Overall, this pseudocode encapsulates a sophisticated exploration strategy, intertwining map updates with complete exploration detection to ensure consistent and accurate representation of the environment. The use of FIFO queues facilitates organized data storage, contributing to the efficiency and adaptability of the exploration algorithm.

#### 2.1.2. Performance Metrics for the Proposed Method

The performance metrics of grid-based graph exploration play a pivotal role in evaluating the effectiveness and efficiency of adaptive robotic-exploration algorithms. Several key metrics are typically employed to assess the performance of such systems. Map complexity measures and acquires data from an algorithm representing an entire environment in a constructed map and is a fundamental metric. The coverage percentage evaluates the proportion of the total environment explored by a robot. Additionally, loop closure detection accuracy is crucial to ensuring the consistency and correctness of a mapped environment, as it indicates an algorithm’s capability to identify and handle revisited locations. Computational efficiency, such as the runtime and memory usage of an algorithm, is essential for real-time applications. These metrics collectively provide a comprehensive view of the performance of an algorithm, guiding the refinement and optimization of grid-based graph exploration strategies for autonomous robotic systems in diverse and dynamic environments.

Activation Rate Statement: For each grid cell (i), the activation rate (R_i_) is the ratio of the number of times the sensor in cell i is activated (A_i_ = 1) to the total number of observations recorded by the sensor.

(4) $$R_{i}=\frac{\text{Number}\;\text{of}\;\text{Activations}\;\text{in}\;\text{Cell}\;A_{i}}{\text{Total}\;\text{Observations}\;\text{by}\;\text{Sensor}\;\text{in}\;\text{Cell}\;i}$$

Claim: If R_i_ is high for each grid cell (i), the system effectively senses the objects in each cell.

Proof: A high activation rate (R_i_ ≈ 1) indicates that the sensor in cell i consistently detects objects, providing evidence of effective sensing.

Grid Coverage Percentage Statement: The percentage of the grid covered (P) is the ratio of the number of grid cells with at least one activation to the total number of grid cells.

(5) $$P=\frac{\sum_{i=1}^{N}A_{i}}{Q}$$

Claim: If P is close to 100%, the ultrasonic sensors effectively covered the entire m × n grid.

Proof: A high percentage (P ≈ 1) implies that almost all grid cells were activated at least once. This suggests comprehensive coverage by ultrasonic sensors.

Completion of Exploration in a Grid:

Objective: Maximize exploration completion (Et).

Time Constraint: Exploration completion (Et) should increase over time but should not exceed 1. Therefore, 0 ≤ Et ≤ 1.

Exploration Progress Constraint: Exploration completion (Et) should reflect the progress made in each time step. Therefore,

Et + 1 ≥ Et.(6)

Interpretation:

The objective is to maximize exploration completion (Et), which represents the overall progress of exploration. The variable Et is constrained between 0 and 1, ensuring a valid completion percentage. The exploration completion at each time step (Et + 1) should be greater than or equal to the completion at the previous time step (Et), ensuring that exploration progresses over time.

Proof Strategy:

Progress Metric: The progress or completion of the grid is measured by the grid coverage percentage. The exploration state (Et) is updated at each time step with the distances received from the sensors (Et + 1 = Et + δ).

Non-negativity: Exploration completion (Et) is always non-negative (0 ≤ Et). Et starts at 0, and δ is a positive value. Therefore, Et + 1 is always non-negative.

Efficiency and Accuracy of a Parallel Algorithm:

T_p_: processing time of the parallel algorithm; M: total memory usage of the parallel algorithm; A: accuracy metric of the parallel algorithm; E: efficiency metric of the parallel algorithm; P: degree of parallelism.

Hypotheses:

Parallel Processing Hypothesis: The processing time (T_p_) of the parallel algorithm is influenced by the degree of parallelism (P) and parallel efficiency (η), where η is a measure of how well the algorithm scales with increasing processors.

T_p_ = h(n, P, η) (7)

Memory Complexity Hypothesis: Memory usage (M) is influenced by the memory complexity of the algorithm, denoted as O(g(n)), and the degree of parallelism (P).

M = k(n, g(n), P)(8)

Accuracy Hypothesis: Accuracy (A) is influenced by the algorithm’s ability to correctly generate output results, considering factors such as true positives and false negatives.

(9) $$A=\frac{\mathrm{TP}+\mathrm{TN}}{\mathrm{TP}+\mathrm{TN}+\mathrm{FP}+\mathrm{FN}}$$

Efficiency Hypothesis: Efficiency (E) is influenced by the parallel efficiency (η), accuracy (A), and resources utilized (T_p_ and M).

(10) $$E=\frac{A}{\alpha T_{p}+\beta M}$$

where α and β are weighting factors representing the relative importance of time and memory efficiency.

### 2.2. Hardware Schemes for the Grid Flex-Graph Exploration Approach

**Figure 4:**
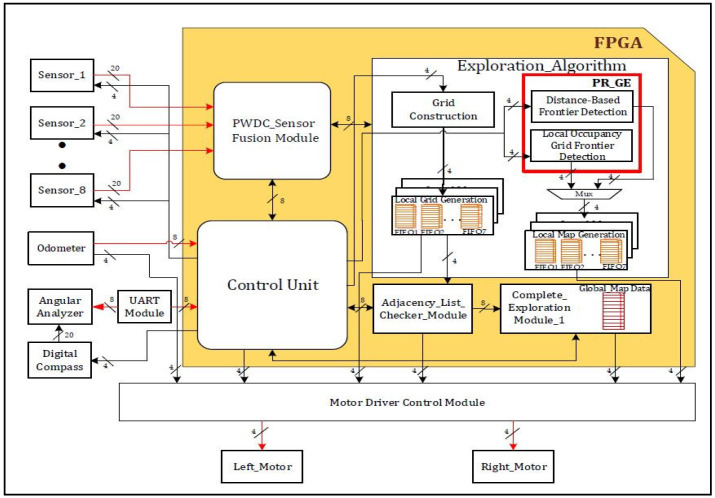
Overall hardware scheme for grid flex-graph exploration method.

The proposed grid-based graph exploration approach is illustrated in Figure 4. Exploration involves the use of ultrasonic sensors and a digital compass to efficiently perceive and navigate a robot’s surroundings and black color arrows shows the data flow sent to the control unit. Sensors use time-of-flight (TOF) to calculate the distances to nearby objects, which are then processed into 20-bit digital data using the pulse width modulation technique for mapping algorithms as shown with red color arrows. In addition, 12 bits are used to represent the sensor ID and the position of the robot. Therefore, 32-bit data are used by the AXI-based 32 × 32 FIFO control module are shown with orange color boxes to drive the output, thereby improving the robot’s perception of the space and providing more precise movement guidance.

The proposed approach was implemented on FPGA as shown in yellow area in an environment with static and dynamic objects. A partial reconfiguration-based frontier dimension was added to the environment, depicting graph exploration by incorporating distance-based or local occupancy in a quadtree structure as shown in red color frame box and arrows represents the flow of data in to fifo. Distance-based frontier detection is based on the distance from a parent node to a child or sibling node. Frontiers are identified as areas near unexplored nodes. The local occupancy grid frontier detection module detects frontiers by analyzing the local occupancy grid cell information generated by the robot’s sensors. Frontiers are identified as boundaries between occupied and unoccupied cells on the local map, as illustrated in Figure 2. This grid, graph, and quadtree combination offers a thorough and flexible method for self-directed exploration and is a novel approach for our system, particularly in situations where a hierarchical understanding of spatial characteristics is essential for effective mapping and navigation.

The adjacency list checker unit is a crucial component in the graph, indicating that the spatial connection is dynamically updated as the robot moves across its neighboring nodes. Its main purpose is to detect complete exploration, check any redundant connections, and cross-reference the connectivity information from the adjacency list with the sensor data from the robot. Through constant observation of the graph’s representation of the workplace, this module supports consistency between the construction of the map and the graph, which is essential for making trustworthy decisions when exploring. To maximize the computational performance and minimize the graph size, this module applies relative-distance strategies.

The complete exploration detection module is a crucial component that helps to identify connectivity among the other vertices in a graph, enabling thorough awareness of the environment of the robot. Its primary function is to detect and handle the completion of exploration loops, ensuring that the robot does not revisit previously explored frontier cells to enhance map consistency. This module employs Algorithm 2, which detects completion by comparing the current sensor data of the robot with the data recorded during its initial exploration. Once a completion is detected, this module triggers appropriate actions, such as refining the map, updating the graph, and merging the local map data to maintain a coherent and accurate representation of the environment. The effective completion of the exploration mechanism contributes to the reliability and completeness of the robot’s mapping and graph construction, enabling it to adapt to changes in the environment and avoid redundant or conflicting data in the exploration process. Stepper motors are used to implement the movements and directional shifts of the robot with an extremely high level of angular accuracy using an odometer during exploration. A stepper motor uses a pulse-width modulation technique to drive a four-bit output from the control module, where each step represents a transfer from a particular grid cell to the next. The processing of the sensor data allows the robot to adhere to the grid structure throughout the exploration and is guaranteed by this controlled mobility. The overall system operates with event-based constraints, using a control unit to synchronize different frequencies and interfaces. The proposed method utilizes partial reconfiguration tools from Xilinx to reduce power consumption and provide efficient synchronization.

#### 2.2.1. Hardware Schemes for Grid Construction and Graph Exploration

**Figure 5:**
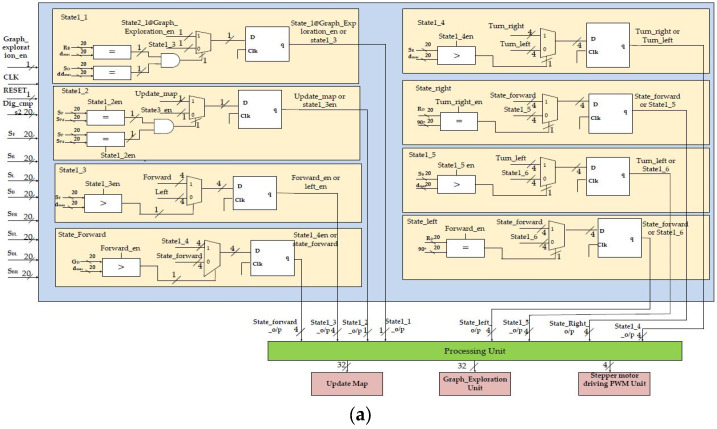

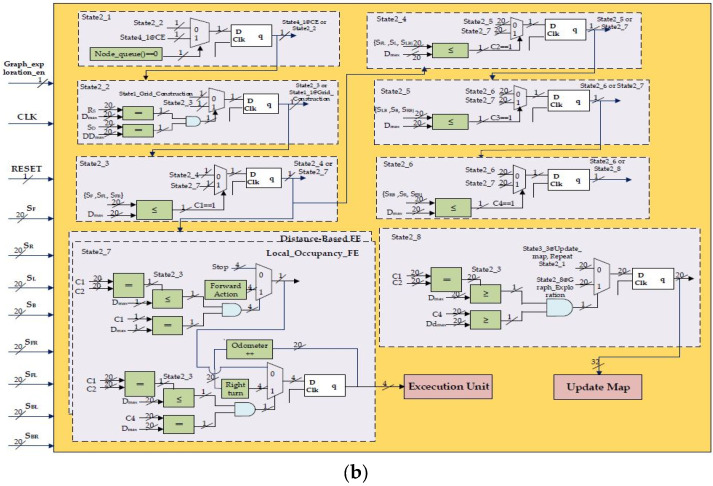
(**a**) Grid construction and internal architecture. (**b**) Graph exploration and internal architecture.

The internal hardware schemes of the proposed approach for grid construction and graph exploration are shown in Figure 5a,b. At its core, the architecture defines the representation of the grid and determines the size, resolution, and methodology for storing information about each grid cell. The robot’s angular values are calculated with a digital compass in real time, whereas the Xilinx CORDIC IP core is used for further digitization based on the reference angle. The quad-grid construction module assesses the robot’s position during grid construction in scenarios that consist of static and dynamic objects. For dynamic objects, the robot adjusts its angular position to a straight line by utilizing real-time digital compass data and CORDIC IP cores to search for neighboring child nodes.

This design incorporates interfaces as shown in light blue color for seamless interaction with ultrasonic sensors, managing the flow of data from the sensors to dedicated control processing units. The connecting nodes and their respective adaptations form the graphs. There are various loops in the system, and the nodes display hierarchical relationships that deviate from conventional graph structures. We introduce the concept of a partial reconfiguration-based tree-like graph structure that includes distance-based or local-occupancy-based frontier information. The proposed method for frontier-graph exploration incrementally expands the graph by identifying and incorporating frontier nodes until no additional nodes are identified. The complete environment represents scattered nodes connected by edges during the graph search operation. These processing units form the computational core and execute algorithms for sensor fusion, occupancy detection, and grid updates. Parallel processing plays a pivotal role in enhancing the speed of grid construction, enabling multiple processing units to operate concurrently. Moreover, VLSI architecture prioritizes power efficiency, which is vital for resource-constrained robotic systems, ensuring that the grid construction process remains synchronized with environmental changes and sensor inputs. Once the exploration of the corresponding grid is complete, the map data are updated. The development of hardware schemes is a novel approach for the proposed method.

#### 2.2.2. Updating Map and Complete Exploration Using Hardware Schemes

**Figure 6:**
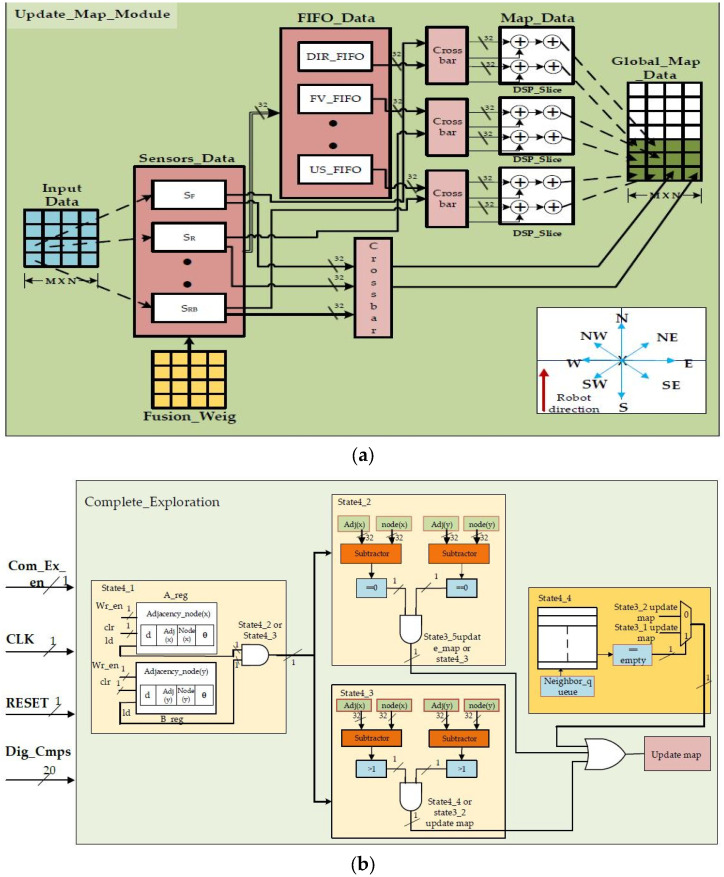
(**a**) Hardware scheme for updating map. (**b**) Hardware scheme for complete exploration.

The internal hardware schemes of the proposed approach for updating the map and complete exploration are shown in Figure 6a,b. The updated map architectures show the data handling methods for different FIFOs used for storing information, and the right side of the figure shows the global map data. LUTs are employed to implement data fusion algorithms and decision-making processes to integrate information from multiple sensors. Initially, local map data streams from various sensors are processed and stored in BRAMs to provide fast and efficient access to the data.

Redundancy checks are performed using DSP slices, which are utilized for computations, such as cross-correlation or similarity measures between data streams from different sensors. DSP slices also enable the real-time processing of sensor data for dynamic redundancy detection and correction, enhancing the reliability of the mapping system. The final vertex, ultrasonic sensor distances, grid information, graph-connecting nodes, dynamic objects, previous history of the map, and direction of the robot are stored. The AXI-based FIFO from Xilinx provides a robust solution for storing relevant information in a grid-based environment requiring a size of 32 × 32 width with a total of eight FIFO instances plus one FIFO instance to store the global map data. By leveraging the advanced extensible interface (AXI) protocol, these FIFOs facilitate efficient communication and data transfer between different processing units within the FPGA design. Each FIFO instance accommodates data from a specific processing unit of the grid, allowing parallel processing and seamless integration of sensor data. With a size of 32 × 32 (depth and width), these FIFOs can store a substantial amount of information, thereby ensuring that the FPGA can effectively manage the memory requirements of complex robotic exploration and mapping tasks. Complete exploration is a crucial state-of-the-art approach for the proposed method. This module re-optimizes the data stored in an FIFO, ensuring that data captured by the robot during traversal do not need to be stored again unless there is a change in the updated data. This is performed by checking the adjacent nodes with the current nodes. If there are no adjacent nodes, this indicates that the robot does not have child nodes to explore in the environment. This comprehensive design approach enables the module to enhance global map consistency by correcting errors that may accumulate during the exploration process, thereby contributing to the robustness and reliability of the mapping system in dynamic environments.

## 3. Results

This section focuses on the proposed findings regarding complete exploration using a grid-based graph approach. This includes discussions of hardware algorithm resource utilization, power analysis, and experimental validation. The FPGA-based accelerators shown in Figure 4, Figure 5 and Figure 6 were created using Verilog HDL, modeled, and synthesized using the Xilinx tool Vivado 2017.4, which was licensed through the Xilinx University Program. A recent survey [30] analyzed FPGA-based robotics and verified the effectiveness of the proposed approach.

### 3.1. Resource Utilization

The proposed approach uses a hardware scheme for robotic exploration in indoor environments with static and dynamic objects. Hardware description language (HDL) was used to describe the functionality of the proposed approach, and the code was converted into its equivalent gate-level netlist, which was synthesized, mapped, and implemented, resulting in configuration bit streams using partial reconfiguration flow, which is a novel approach. These bit streams were loaded onto an SD Card to ensure compatibility with the FPGA programming process. When loaded onto the FPGA, this bit stream configured the hardware to execute partial reconfiguration-based frontier exploration algorithms during the runtime [31,32]. An FPGA operated at 100 MHz was used to perform computations with other devices operated at lower frequencies.

Zed board bearing part No. XC7Z020, developed by Xilinx, a company based in San Jose, CA, USA, was used to develop the algorithms and the control module. This device has approximately 85 K programmable logic cells that incorporate a lookup table (LUT) and flip-flops. These components are utilized for logic operations and short-term memory storage. Block RAMs (BRAMs) in field-programmable gate arrays (FPGAs) are versatile on-chip memory resources responsible for the creation of memory hierarchies within FPGAs, optimizing data storage and retrieval for enhanced performance. These memory blocks offer configurability in terms of size, port modes, and access timings and operate efficiently with low power consumption, providing fast and direct access to stored data without the need for external memory components. In this work, each block was 36 kb, and there were 140 blocks (3.6 Mb). They were utilized for the storage of sensor fusion data and intermediate data storage, as most FIFOs used in the proposed approach used BRAMs. DSP slices were utilized for internal computations, including data sharing between the temporary registers and memory. The number of available DSP slices was approximately 220 for each slice, including 18 × 25 multiply–accumulate units (MACCs). The resources used to execute the proposed approach are listed in Table 2.

Zed board has a limited number of FPGA resources compared with other FPGA boards. We endeavored to enhance this method to obtain cost-efficient solutions. The experiment was conducted in two ways as follows: in an environment with dynamic objects and in an environment with static and dynamic objects. Overall, the device utilization with partial reconfiguration flow for the approach was 57.71% (30,707), 77.14% (108), and 70.90% (156) for the LUTs, BRAM, and DSP slices, respectively, as listed in Table 2. At the same time, the static power consumption was approximately 1.8 watts with respect to device utilization. According to the authors of [24], the acceleration of the updating of map data was achieved by employing an extensive pipeline that enabled one cell to be updated every clock cycle. Furthermore, the dual-port map memory lacked the capability to increase parallelism because it required a read operation to be executed in each cycle. In addition to the write operation, and considering the tradeoff between cost and speed, the proposed method addressed the quadtree data updated on the map per clock cycle. As per the authors of [7], the FPGA resources utilized only for the implementation of backtracking occupancy grid-based mapping were 4581 LUTs, using a part of the 32 KB of RAM and a part of the 512 KB of external RAM. Hence, the novelty of the proposed approach is that it utilized on-chip memory available in the form of BRAMs to optimize the design while handling the mapped data. Table 3 shows a summary of the comparative data of the traditional methods utilized in terms of device resource utilization.

**Figure 7:**
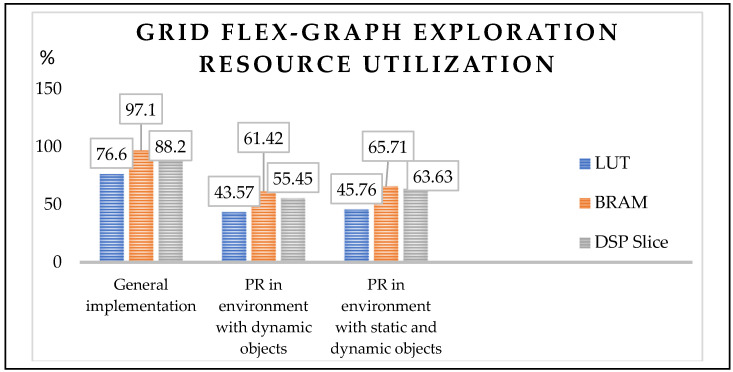
Proposed approach: resource utilization in an indoor environment.

Figure 7 provides a quantitative comparison of device utilization in an environment that consists of dynamic and static objects. A Xilinx-based Integrated Logic Analyzer (ILA) was employed as a tracking tool to assess devices that employ individuals while implementing the proposed approach.

**Figure 8:**
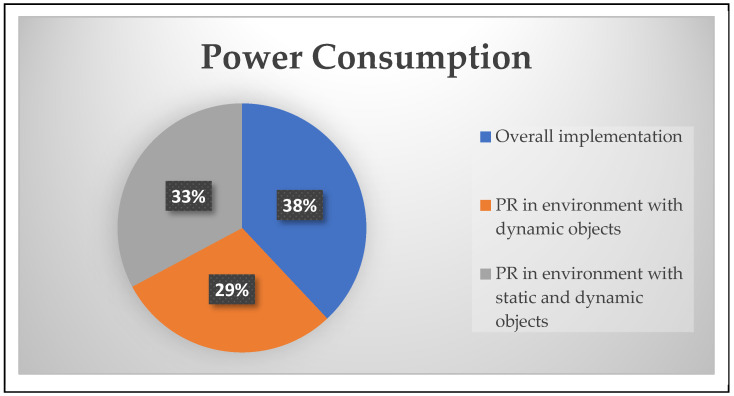
Device power consumption for proposed approach in an indoor environment.

The power consumption of the LUTs, BRAM, and DSP slices was 43.57%, 61.42%, and 55.45% in an environment with dynamic objects and 45.76%, 65.71%, and 63.63% in an environment with static and dynamic objects, respectively. Similarly, the static power consumption with partial reconfiguration flow by the above modules was monitored using a Xilinx Power Estimator (XPE). As illustrated in Figure 8, values of 1.69 watts and 1.92 watts were recorded.

### 3.2. Experimental Results

**Figure 9:**
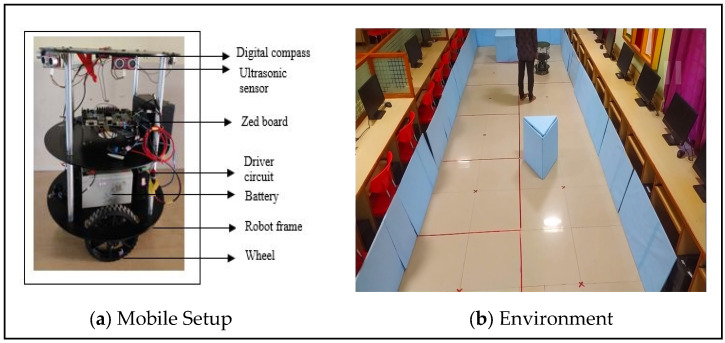
(**a**,**b**) Experimental setup of the mobile robot.

The following section focuses on experimentally verifying the validity of the proposed method, as shown in Figure 9a. During the validation phase, we created test environments using a mobile robot, as shown in Figure 9b.

#### 3.2.1. Experimental Setup

The mobile robot was integrated with mechanical, electrical, and computational devices. Eight ultrasonic sensors were placed 45° from the positions of the sensors. The robot was supplied with a 24-volt, 7 amp battery. The supplied battery voltages were dropped to 5 V using voltage regulator modules, which served as a power supply for the electrical devices and computing equipment. Stepper motors were positioned symmetrically on both sides of the robot’s frame. The structure was composed of many sections, with the bottom one holding the integrated stepper motors, the next level accommodating the batteries, and the level above that containing the electrical components and the computational device. The top surfaces of the sides were linked to the sensors and a digital compass.

#### 3.2.2. GFGE Experimental Results in an Environment with Dynamic Objects

**Figure 10:**
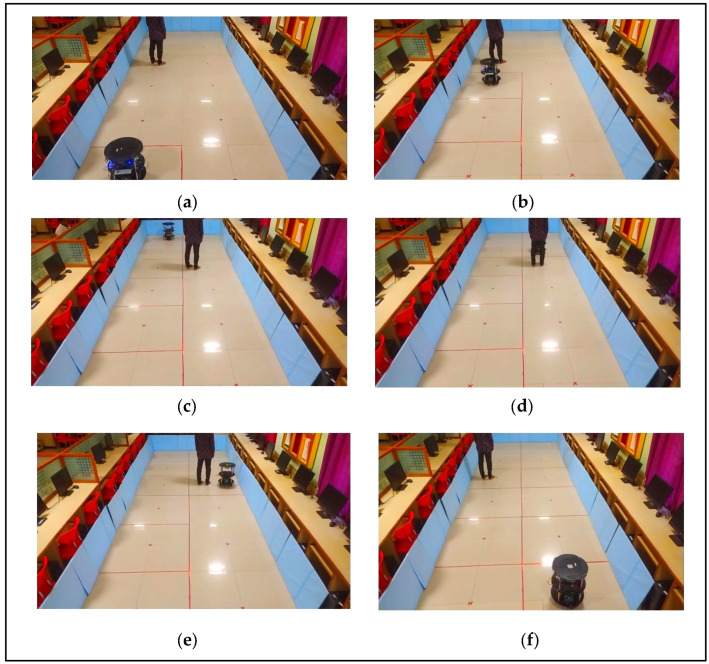
(**a**–**f**) GFGE Empirical results of robot exploration in an environment with dynamic objects.

The GFGE was validated in an environment with dynamic objects, as shown in Figure 10a–f, and the hardware schemes for dynamic changes in the environment are shown in Figure 4. Initially, the robot localizes itself by constructing the grid to reach the root node of the grid (R), as shown in Figure 10a with red color lines. It then commences exploration utilizing information from neighboring grid cells, facilitated by the partial reconfiguration module. In this scenario, a quadtree structure was established, with the root node representing the entire grid (R) and the frontier cells of the environment (NW, NE, SW, and SE). The next neighboring child node or sibling node is selected by a distance-based frontier detection module or a local-occupancy-based frontier detection module, which dynamically switches its behavior during runtime with the help of PR flow. As the robot encounters dynamic elements, such as a moving human, represented as dynamic objects, it dynamically adapts its behavior by analyzing changes in the occupancy cells of the quadtree nodes. For example, upon encountering a dynamic human within a specific quadrant, the robot adjusts its path by selecting a different frontier cell within the same quadrant or exploring neighboring quadrants, as depicted in Figure 10a–c. This flexible behavior is facilitated by recursively traversing the quadtree structure to identify suitable exploration paths while avoiding objects. When the robot returns with the help of adjacency nodes in Figure 10d, the human changes its position, and the robot confines the dynamic object, as illustrated in Algorithm 1, updates the equivalent information on to the map, and takes other directions where the occupancy states are empty. As the exploration progresses, the robot continuously updates the occupancy information in the quadtree to reflect the changes caused by dynamic objects. When a human changes its position, the robot updates the occupancy states of the relevant quadtree nodes and redirects its exploration towards the unoccupied cells, as illustrated in Figure 10d. Therefore, after crossing the human, the robot will again analyze to be at the center of the grid node and complete its exploration, as shown in Figure 10e,f. The experimental presentation was uploaded to the YouTube channel https://youtu.be/ZGxsBRnqeB0 (accessed on 23 February 2024).

#### 3.2.3. Experimental GFGE Results in an Environment with Static and Dynamic Objects

**Figure 11:**
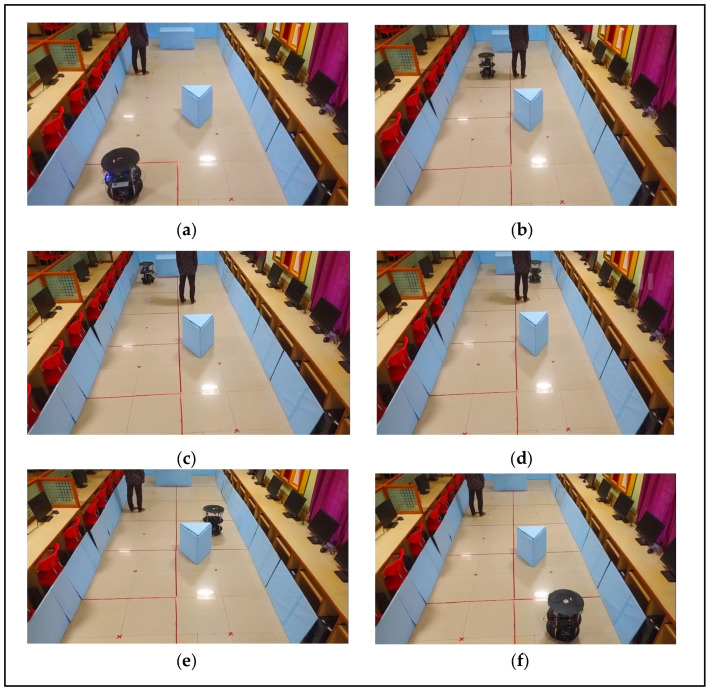
(**a**–**f**) Empirical GFGE results of robot exploration in an environment with static and dynamic objects.

The GFGE was validated in an environment with static and dynamic objects, as shown in Figure 11a–f, and the hardware schemes for the dynamic changes in the environment are shown in Figure 5. When there were static objects, such as stationary boxes, or dynamic objects, such as humans, in the environment, the robot needed to store the occupancy state information every time it encountered the objects, as shown in Figure 11a–f. As stated for exploration with dynamic objects, the robot updated the status of the frontier free cell in the grid map to construct a quadtree structure, as shown in Figure 11a,b with red color lines. For instance, when the robot encountered a static object, based on sensor fusion data, it analyzed the free cell of NW, NE, SW, or SE and switched to a local-occupancy-based frontier detection approach. The robot, as shown in Figure 11c,d, overcame the object. Once the robot crossed the dynamic object, it again came across the static object, and the same approach was followed to overcome it. The robot completed its exploration by avoiding the objects, as shown in Figure 11e,f. An experimental presentation was uploaded to a YouTube channel at https://youtu.be/ZGxsBRnqeB0 (accessed on 23 February 2024).

Table 4 lists the relevant fields of the grid flex-graph exploration approach. Most researchers have used laser range finders to obtain sensor data, but they have not specified how to sample and filter the noisy information received from sensors [3,4]. Frontier-based or topological approaches have been adopted for robot exploration [3,14], but partial information must be loaded before the robot starts exploration. In [9,16], the authors addressed the loop closure problem, but the selection of the optimal direction for a robot is not defined, which may lead a robot to traverse the same node in a graph. Therefore, it requires large amounts of computational analysis and power. The error rate of the proposed algorithm was calculated based on experiments conducted under various environmental conditions. The performance results of these experiments are listed in Table 5. The central focus of the proposed approach is the identification of static and dynamic objects within a grid-based structure and the decision to traverse neighboring nodes. This resulted in a reduction rate of 0.8%. Distance-based frontier detection and a local occupancy grid frontier detection graph approach were utilized for the robot to select the next neighboring node to visit for exploration using the partial reconfiguration method. This resulted in a reduction rate of 1.6%. The performance metrics include the objective function, the percentage of the environment a robot has explored, and connectivity between nodes and edges with redundancy checks during the exploration process, as shown in (1) to (6). Overall, based on related work and this comparison, few researchers have contributed to robotic exploration systems by adopting individual or adaptive algorithms for the fast completion of exploration. The proposed approach provides a good solution for grid flex-graph exploration in an environment that consists of static and dynamic objects with PR flow. Map information is updated simultaneously to carry out complete exploration quickly via fast computations using hardware devices and equivalent hardware schemes.

## 4. Conclusions

In conclusion, the grid flex-graph exploration (GFGE) approach has emerged as a groundbreaking and adaptive methodology for single-robot mapping in grid-based environments. The seamless integration of quadtree structures, coupled with the implementation of ultrasonic sensors, enables GFGE to both capture local details and maintain global map consistency, thereby improving dynamic graph optimization. The parallel processing capabilities of an FPGA enhance computational efficiency, making GFGE well suited for real-time applications. The incorporation of a complete exploration module adds a layer of sophistication, allowing the algorithm to detect revisited locations and refine map consistency over time. The algorithms were coded using a hardware description language, and their VLSI architectures were represented. The performance metrics included the resource utilization of the algorithm in an environment with dynamic objects (43.57%, 61.42%, and 55.45%) and an environment with static and dynamic objects (45.76%, 65.71%, and 63.63%, respectively). The power consumption of the device was 1.69 and 1.92 watts, respectively. The future trajectory of GFGE holds considerable promise, with avenues for further optimization of data handling and the integration of additional sensors to increase complete exploration by multi-robotic systems. As hardware advances continue, algorithms are poised to embrace emerging technologies, ensuring their relevance and effectiveness in reshaping the landscape of autonomous exploration and mapping in diverse real-world scenarios.

## Figures and Tables

**Table 1:** Proposed abbreviations pertaining to our research work.

Symbol	Symbol Description
R_S_	Robot sensors facing 90° angles {S_F_, S_L_, S_R_, S_B_}. F: front, L: left, R: right side, B: backward.
S_D_	Robot sensors facing angles 45° from each R_S_ {S_FR_, S_FL_, S_BL_, S_BR_}. FR: front-right, FL: front-left, LB: backward-left, RB: backward-right.
R_P_, F	Robot position (x_i_, y_i_, θ_i_) and memory required to store the data {7{R × C}}.
E	Unknown environment is represented in a 2D space with a boundary limit of M × N. There are objects whose positions are not known.
D_max_ DD_max_	Maximum distance in robot’s vicinity. Maximum diagonal distance in robot’s vicinity.
d_max_ dd_max_	Maximum distance in grid’s vicinity. Maximum diagonal distance in grid’s vicinity.
d_min_ dd_min_	Minimum distance in grid’s vicinity. Minimum diagonal distance in grid’s vicinity.
M_ij_	The occupancy state of cell (i, j) in the grid. M_ij_ = 1 indicates the presence of an object, and M_ij_ = 0 indicates a free space. This binary matrix represents the occupancy map.
G_ij_	The graph representation of the grid. G_ij_ = 1 indicates a connection between nodes (i_1_, j_1_) and (i_2_, j_2_), and G_ij_ = 0 indicates no connection. This graph dynamically evolves based on sensor readings and completion detection.
D_i_	Ultrasonic distance readings from the eight sensors. These variables provide information about the distances in the robot’s vicinity between the node and surrounding neighboring nodes.
FD_max_, FD, FG, FV, FO, FMH, PMH	Final maximum distance, final direction, final grid, final vertex, final object, final map history, and previous map history, respectively.
Q, m × n	Number of grid cells and the size of the grid represented as a quad-grid.
Et	Exploration completion at time step t (a value between 0 and 1, where 1 represents no exploration and 0 represents complete exploration).

**Table 2:** Zed board FPGA resource utilization for grid flex-graph exploration.

Module	LUT	BRAM	DSP Slice
Communicating modules (sensors, motors, UART, and Xilinx IP cores)	6552	20	38
Control unit and PWDC sensor fusion	4686	18	32
Complete exploration module	5586	32	36
PR in environment with dynamic objects	6358	16	16
PR in environment with static and dynamic objects	7525	22	34
Total	30,707	108	156

**Table 3:** Comparative data of traditional methods for resource utilization.

Reference No.	Methods Used	Computing Device	FF	Four-Input LUT	DSPs	Internal RAM	External RAM	Reconfigurable Feature
[7]	Backtracking occupancy grid-based mapping	FPGA Xilinx Spartan3-1000 (Xilinx, Hyderabad, India)	3228	4581	18	32	512	No
[24]	Scan-matching genetic SLAM	Xilinx Virtex-5 (Xilinx, Hyderabad, India)	2377	4573	9	131	Not used	No
Proposed approach	Grid flex-graph exploration	Xilinx Zed board FPGA (Xilinx, Hyderabad, India)	4686	6358, 7525	16, 34	16, 22	Not used	Yes

**Table 4:** Comparison of grid flex-graph exploration and previous research approaches.

Reference Papers	Sensory Approach		Algorithm	Hardware	Pros	Cons
	Method	Fusion				
[3]	Sensor data	X	Frontier-based graph approach	CPU	Rapidly encompasses the area with its sensors beyond the capabilities of a greedy exploration system.	Need to provide additional information before exploration.
[4]	Laser scans	X	Topological feature graph	CPU	Vertices and edges can be learned using the depth of the image or laser scans.	Limited to traversing the edges of graphs.
[9]	Sensor perception data	X	Single immovable directional marker	CPU	Loop-closing problem in embedded topological worlds using an external marking aid.	Selection of an optimal location for the marker is not defined, the computational cost is high, and this method is limited to simulation.
[14]	Sensor data	X	Predictive exploration approach	CPU	The structures of environments in unexplored areas are obtained using previous data.	Utilizing prior knowledge while performing autonomous operation.
[16]	2D laser range finder	X	Safe and Reachable Frontier Detection (SRFD)	CPU	Data structure for efficient frontier extraction and management.	Need for large computational analysis and power consumption.
Proposed approach	Ultrasonic sensor data	√	Grid flex-graph exploration in static and dynamic environments	FPGA	Grid construction and graph exploration are novel approaches to compute loop closure and VLSI architectures.	Optimization of data handling for different sensors to compute loop closure for multi-robotic systems will be integrated in the near future.

X—Not available √—Available.

**Table 5:** Qualitative comparison of grid flex-graph exploration and relevant research methods.

Map-Type	Ultrasonic Sensor Data Fusion	Sensor Data Fusion Positive Rate	Error Rate
Topology–grid hybrid map/occupancy grid map	Environment with static and dynamic objects	97.2%	2.4%
	PR in environment with dynamic Objects	98.8%	1.6%
	PR in environment with static and dynamic objects	99.4%	0.8%
Topo-metric graph/graph-based representation	Environment with static and dynamic objects	96.2%	3.6%
	PR in environment with dynamic Objects	97.2%	2.2%
	PR in environment with static and dynamic objects	98.6%	1.6%

## Data Availability

Data are available for the experimental validations in the dynamic environment at https://youtu.be/ZGxsBRnqeB0 (accessed on 23 February 2024).
